# The Functional Roles of ISG15/ISGylation in Cancer

**DOI:** 10.3390/molecules28031337

**Published:** 2023-01-31

**Authors:** Yin Yuan, Hai Qin, Huilong Li, Wanjin Shi, Lichen Bao, Shengtao Xu, Jun Yin, Lufeng Zheng

**Affiliations:** 1 Jiangsu Key Laboratory of Carcinogenesis and Intervention, Department of Medicinal Chemistry, School of Life Science and Technology, China Pharmaceutical University, 639 Longmian Road, Nanjing 211198, China; 2 Department of Clinical Laboratory, Guizhou Provincial Orthopedic Hospital, No. 206, Sixian Street, Baiyun District, Guiyang 550002, China; 3 Jiangsu Key Laboratory of Neurodegeneration, Nanjing Medical University, Nanjing 210029, China

**Keywords:** ISG15, ISGylation, cancer, ubiquitin-like protein, post-translational modification

## Abstract

The protein ISG15 encoded by interferon-stimulated gene (ISG) 15 is the first identified member of the ubiquitin-like protein family and exists in the form of monomers and conjugated complexes. Like ubiquitin, ISG15 can mediate an ubiquitin-like modification by covalently modifying other proteins, known as ISGylation. There is growing evidence showing that both the free and conjugated ISG15 are involved in multiple key cellular processes, including autophagy, exosome secretion, DNA repair, immune regulation, and cancer occurrence and progression. In this review, we aim to further clarify the function of ISG15 and ISGylation in cancer, demonstrate the important relationship between ISG15/ISGylation and cancer, and emphasize new insights into the different roles of ISG15/ISGylation in cancer progression. This review may contribute to therapeutic intervention in cancer. However, due to the limitations of current research, the regulation of ISG15/ISGylation on cancer progression is not completely clear, thus further comprehensive and sufficient correlation studies are still needed.

## 1. Introduction

Post-translational modifications (PTMs) are important for protein activities because they modulate structure and function by changing properties such as charge, hydrophilicity/hydrophobicity, and spatial structure [1].The forms of protein PTMs are various, including phosphorylation [2], ubiquitination [3], methylation [4], acetylation [5], etc. In recent years, the ubiquitin-like (UBL) modification of proteins has been extensively studied. As a PTM similar to ubiquitination, the proteins mediating UBL modification are structurally and evolutionarily related to ubiquitin. The binding of these UBL proteins to target proteins is also dependent on the level of E1 activating enzymes, E2 binding enzymes, and E3 ligases. The PTM function of UBL proteins is implicated in the nervous system [6], endocrine system [6], cardiovascular system [7], and many other diseases. Currently, the role of UBL modification in tumor occurrence and development is receiving a lot of attention [8,9,10] (Table 1).

**Figure 1:**
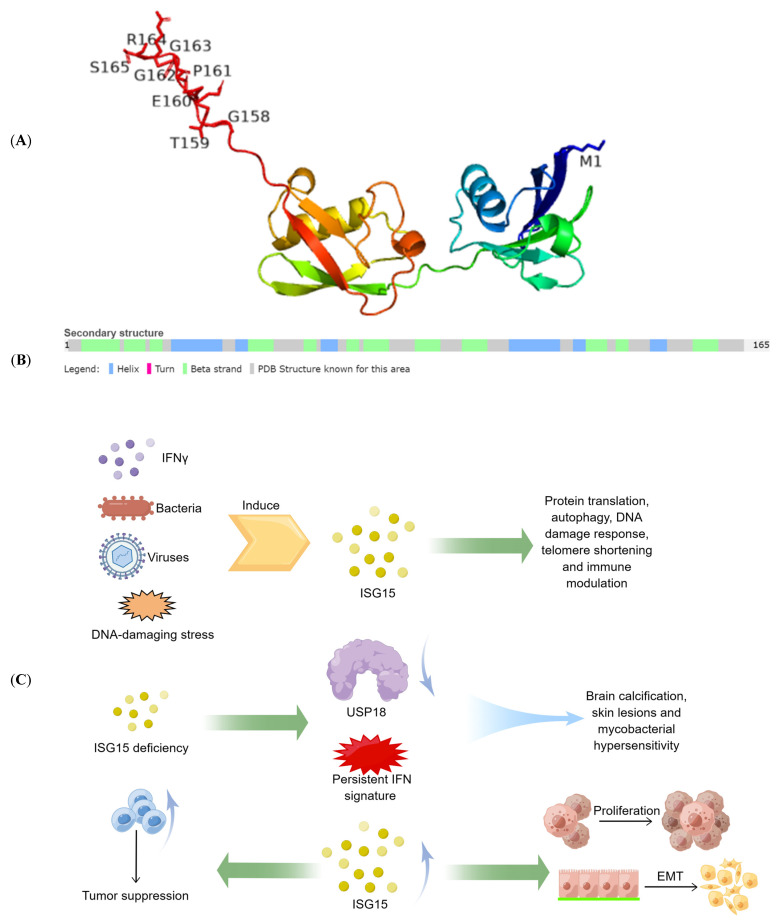
**The structure and function of ISG15.** (**A**) the 3D representation of the ISG15 protein. (**B**) the secondary structure of the ISG15 protein. (**C**) physiological and pathological functions of ISG15. Include the role of ISG15 in antiviral and immune regulation, the harm of ISG15 defects, and the role of ISG15 in tumor proliferation and tumor immunity.

ISG15 is the product of interferon-stimulated gene 15, and its expression is regulated by the interferon signaling pathway. ISG15 was first discovered as an UBL protein in 1979 by Farrel et al. [23]. The precursor to ISG15 has a total of 165 amino acid residues. Through protease splicing and processing, the N-terminal methionine and the C-terminal 8 amino acid residues (GTEPGGRS) are removed, and a mature protein containing 156 amino acid residues is finally formed (Figure 1). ISG15 is an UBL protein with the ability to form covalent protein-binding [24,25]. Although ISG15 and ubiquitin share similarities in sequence and structure, their functions are not exactly identical. In addition to its role in protein binding, ISG15 exists both intracellularly and extracellularly in free form. In recent years, several studies have shown that both ISG15 and its covalently bound form play an important role in various cancers and therapeutic interventions [14,26].

ISG15 can be detected in various kinds of cells, such as lymphoid cells, striated and smooth muscles, epithelial cells, neurons, neutrophils, monocytes, and lymphocytes [14]. In addition, ISG15 expression is increased in pancreatic, endometrial, and bladder cancers compared to that in non-cancerous tissues [27]. Furthermore, ISG15 expression has been found to be significantly positively associated with advanced stages of bladder cancer [28,29].

Unlike ubiquitin, which is highly conserved, ISG15 varies widely across species, with only 50% average similarities to each other. Both UBL domain 1 and UBL domain 2 are required for ISG15’s efficient binding to cellular proteins, while these two domains hold distinct roles during ISGylation: UBL domain 2 is essential for the first two steps of ISG15 attachment to E1 and E2 enzymes; UBL domain 1 is the final step of ISG15 by E3 necessary for lysine-mediated transfer from E2 to target proteins [30].

## 2. Free ISG15 in Cancer

**Figure 2:**
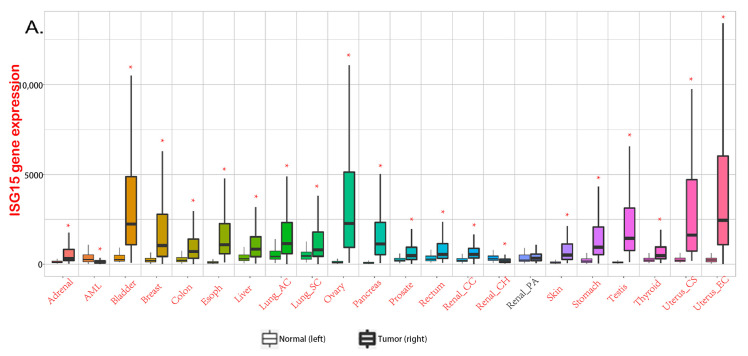

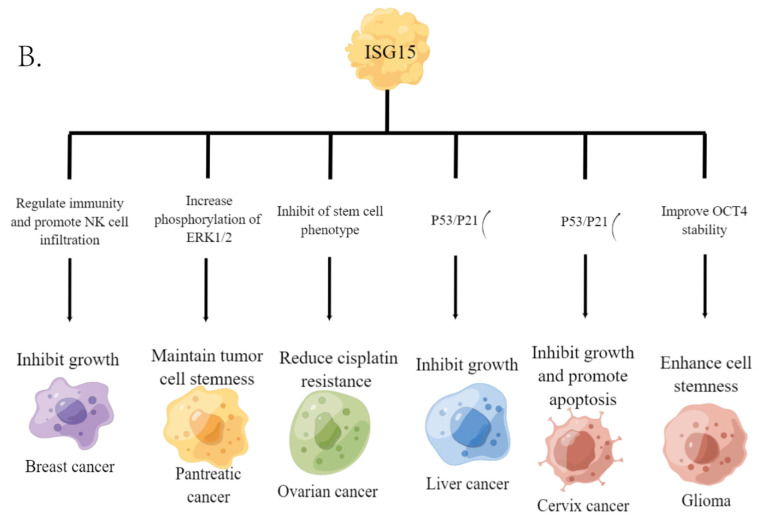
**ISG15 in various cancers.** (**A**) expression levels of ISG15 in different cancers. Data from TNMplot, significant differences by Mann-Whitney U test are marked with red [32]. * *p* < 0.05 vs. normal group. (**B**) the functional roles of free ISG15 in various cancers.

Numerous studies have shown that the production of ISG15 monomer can be stimulated by the activation of the innate immune system [31]. For example, type I interferon can induce cells to express ISG15 in large quantities. Notably, ISG15 displays different levels in different cancers (Figure 2A) [32] and plays a key role in the occurrence and development of tumors. Current studies have shown that ISG15 could promote or inhibit cancer progression in different cancers; for example, free ISG15 can regulate immunity and promote NK cell infiltration, thus promoting breast cancer growth. Furthermore, mammary tumors had higher levels of ISG15 mRNA and protein than normal mammary tissue, and ISG15 may regulate cytoskeleton reorganization [33,34,35]. In pancreatic cancer, free ISG15 may be implicated in maintaining the stemness of tumor cells by increasing the phosphorylation level of ERK1/2 [36]. In ovarian cancer, free ISG15 can inhibit the phenotypes of cancer stem cells (CSCs) to attenuate cisplatin resistance [37]; in liver cancer and cervical cancer, free ISG15 can promote the upregulation of p53/p21 expression, thereby inhibiting tumor progression [38,39]; and in glioma, free ISG15 could improve the stability of OCT4 to enhance the stemness of cells [40] (Figure 2B).

### 2.1. Free Extracellular ISG15

Free ISG15 has been reported outside of numerous cell types, including human primary monocytes, lymphocytes, neutrophils, and plasmablasts with type I interferon (IFN) treatment. ISG15 is also detected in the serum of patients receiving IFNβ treatment as well as patients with hepatitis B virus (HBV) infection [26]. In addition to its chemoattractive properties on murine neutrophils, free extracellular ISG15 also functions as a cytokine, enhancing the cytotoxicity of LPS-stimulated primary monocytes, IFNγ production, and induction of natural killer (NK) cells [29,41,42,43,44]. These results demonstrate that free ISG15 is tightly involved in antiviral ability and immune regulation, which suggests that cancer progression regulated by free extracellular ISG15 may rely on immune-related pathways, such as the interferon pathway [14].

Using inhibitors of the cellular secretion process, D’Cunha et al. proposed that ISG15 is not secreted by the Golgi complex of the canonical protein transport pathway but by the multidrug-resistant glycoproteins in the non-canonical pathways. These data led the authors to link ISG15 to the proinflammatory cytokine interleukin 1β (IL-1β) [45]. Like IL-1β, ISG15 is synthesized from a larger precursor [46], lacks a secretion signal peptide [47], and is not secreted by the classical pathway. Notably, neutrophil granules [47] and microvesicles [48] have been reported as the most likely potential secretory pathways. Extracellular-free ISG15 has been reported to play an important role in various cancers. Previous studies have demonstrated that ISG15 secretion is increased in M2 or activated macrophages, which is tightly associated with tumor growth as well as angiogenesis, tissue remodeling, and suppression of adaptive immunity [49,50]. Studies by Burks et al. demonstrated that extracellular ISG15 inhibited the growth of breast cancer cells and enhanced the migration of NK cells to tumor tissues [51]. Huggins et al. demonstrated that some macrophages in the murine breast cancer microenvironment hold enhanced expression of ISG15-related genes [49]. In addition to breast cancer, there are similar studies in pancreatic ductal carcinoma (PDAC), but they show the opposite conclusion. Sun et al. have proved that extracellular free ISG15 plays an important role in maintaining the CSC-like characteristics of PDAC [52]. The function of extracellular free ISG15 is significantly related to the immune function of the body, which is also an important reason why it is regarded as a cytokine. It can be predicted that the combination treatment targeting the tumor microenvironment and tumor is likely to be a good clinical treatment.

### 2.2. Free Intracellular ISG15

Intracellular free ISG15 has been shown to non-covalently bind to intracellular proteins, thereby regulating the function of its interacting partners. The results show that ISG15 interacts with the NEDD4 ubiquitin ligase, disrupting its ligase activity and resulting in less subsequent ubiquitination of VP40, implying that free ISG15 has antiviral activity by inhibiting the E3 ligase activity of the host NEDD4 [53]. Interestingly, it has been reported that the stabilization of USP18 by ISG15 is independent of its binding ability, and stabilization of USP18 by type Ⅰ IFN-induced ISG15 is essential for USP18-mediated regulation of interferon signaling in a negative feedback loop, leading to prevention of autoinflammation [54].

The regulation of intracellular monomeric ISG15 on tumor cells has also been reported. Burks et al. found that intracellular ISG15 monomer can increase the expression of MHC on the surface of breast cancer cells, exerting anti-tumor properties [51]. Furthermore, high mRNA and protein expression of ISG15 is associated with lymphovascular invasion (LVI), higher histological grade, larger tumor size, hormone receptor-negative, HER2-positive, and HER2-enriched breast cancer subtypes [55]. In addition, Sun et al. indicated that free ISG15 promotes the CSC-like features of PDAC; this effect is regulated by TRIM29 and CAPN3 [53].

## 3. ISGylation in Cancer

**Figure 3:**
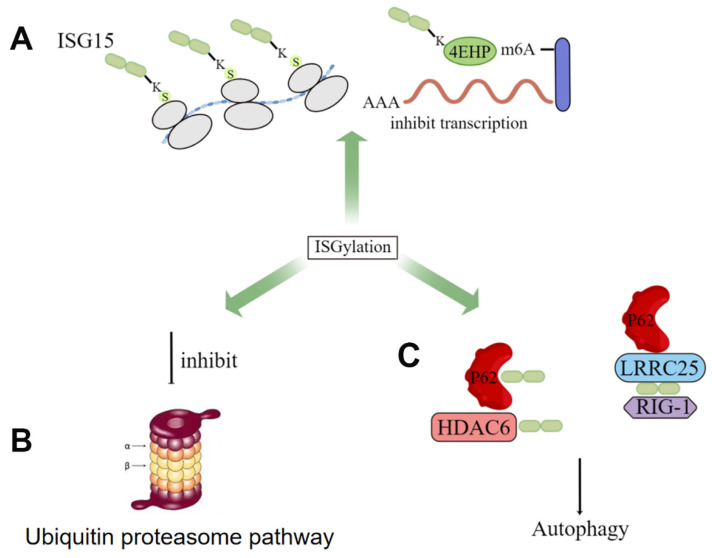
**The role of ISGylation in cells**. (**A**) inhibition of transcription by increased ISGylation levels of some proteins; (**B**) antagonism of ISGylation to the ubiquitin proteasome pathway; (**C**) ISG15 promotes p62-mediated autophagy.

Currently, numerous molecules have been proven to be ISGylated, such as 4EHP [56], protein kinase R (PKR) [57], NEMO [58], human papillomavirus (HPV) L1 capsid protein [59], etc. ISGylation has been proven to be widely involved in human immunity and tumor development. In terms of its underlying mechanisms, although ISG15 shares similar structural and mechanistic features with ubiquitin, the biological consequences of their binding to cellular proteins (ISGylation and ubiquitination) are different. Ubiquitination (Lys48 bond) targets proteins for degradation, while Desai et al. first demonstrated that elevated ISGylation antagonizes the ubiquitin pathway by inhibiting polyubiquitination, and this constitutive elevation of ISGylation mediates the pathogenesis of tumorigenesis [60]. Several groups have now demonstrated that ISG15 inhibits polyubiquitination by regulating the activity of selected ubiquitin E2 and E3 ligases and subsequently inhibiting the degradation of ubiquitin substrates [14]. Besides, some studies have shown that ISGylation can also regulate autophagy by orchestrating autophagy-related proteins, such as ATG5 and Beclin1, and transcription by modulating transcriptional factors, respectively. Specifically, the deletion of ISG15 leads to impaired recruitment of p62, NDP52, and LC3 in parasitophorous vacuoles (PV), resulting in interferon-induced autophagy damage. And ISGylation can further inhibit autophagy by reducing the stability of the proautophagy mediator Beclin1 to inhibit its activity [56,61,62] (Figure 3).

### 3.1. ISG15 and Its Covalent System

**Figure 4:**
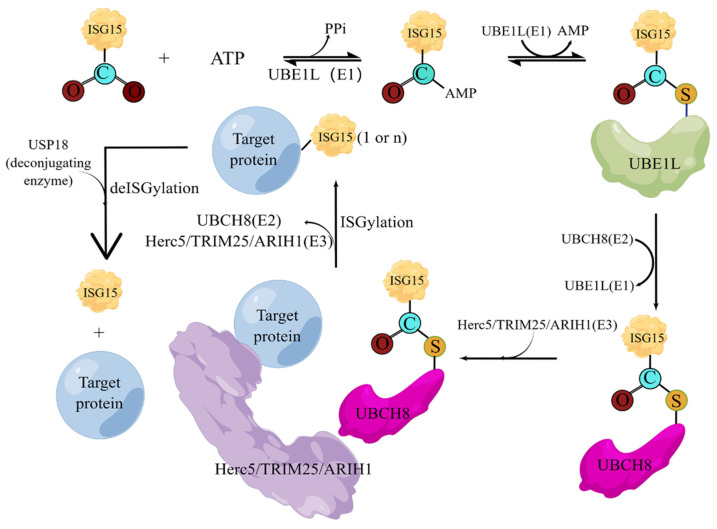
**The cascade reactions of ISGylation.** Under the action of activating enzyme E1, the C-terminal of ISG15 and the active site cysteine (Cys) of E1 form a high-energy thioester bond, and this step requires ATP to provide energy; the activated ISG15 is then transferred to the conjugated enzyme; the cysteine of E2 is also covalently linked in the form of a thioester bond; and finally, under the action of ligase E3, ISG15 is modified on the substrate protein. The deconjugating enzyme ISG15 can enzymatically hydrolyze ISG15 from the modified substrate, thus forming ISG15 recycling.

Like ubiquitin, the covalent modification of ISG15 also requires the E1 activating enzyme UBA7 [63], the E2 binding enzyme UBCH8 [64], the E3 ligase HERC5, ARIH1 [65], and TRIM25 [66]. The entire ISGylation is also an enzymatic cascade reaction. E1 uses the energy provided by ATP to generate the ISG15-E1 complex. The ISG15-E1 complex transfers ISG15 to E2 through transesterification to form the ISG15-E2 complex, and then ISG15 is transferred to E3 through transesterification, and finally E3 specifically recognizes the target protein and links ISG15 to the Lys residue of the target protein. Meanwhile, the level of intracellular ISGylation is also affected by the de-ISGylase USP18 [67], which can specifically cleave ISG15 fusion proteins, including native ISG15 conjugates linked by isopeptide bonds, which are responsible for maintaining ISG15 in healthy and stressed organisms and necessary for key cellular homeostasis of binding proteins (Figure 4) [68].

### 3.2. ISGylation Level and Cancer Progression

**Figure 5:**
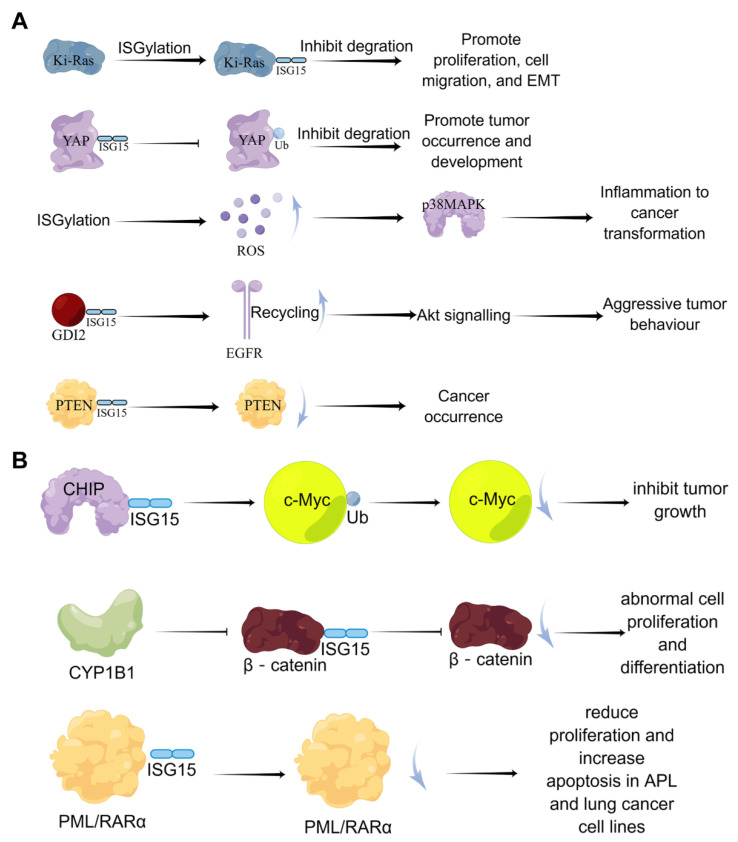
**The dual-function of ISGylation in tumor cells.** (**A**) the cancer-promoting effects of ISGylation; (**B**) the tumor-suppressive effects of ISGylation.

Currently, the effects of ISGylation on cancer seem to be multi-faceted. A large number of studies have shown that ISGylation exerts both tumor-promoting and tumor-suppressive effects in tumor cells, which depend on tumor types (Figure 5). Burks et al. demonstrated that ISGylation inhibits the degradation of Ki-Ras, thereby promoting cell proliferation, migration, and epithelial-mesenchymal transition (EMT) [69]. Oncogenic Ki-Ras regulates the expression of ISG15 and ISGylation, which in turn stabilizes Ki-Ras by inhibiting its degradation through lysosomes in breast cancer cells, suggesting that the ISGylation pathway is a key downstream mediator of oncogenic Ki-Ras. Also, it has been reported that ISGylation drives basal breast tumor progression by promoting EGFR recycling and Akt signaling [70].

In addition, Xue et al. found that in lung cancer cell lines, Yes-associated protein (YAP) can undergo ISGylation, and this ISGylation inhibits YAP ubiquitination, proteasomal degradation, and interaction with beta-transducin repeats containing E3 ubiquitin-protein ligase (βTrCP) to promote YAP stability, thereby promoting the occurrence and development of tumors [71]. In prostate cancer cells, inhibition of covalent modification of ISG15 suppressed cell proliferation [72]. In contrast, ISGylation of the tumor suppressor gene PTEN can reduce its stability and tumor-suppressive ability [73]. The covalent modification of ISG15 also negatively regulates the ubiquitin-protease system to generate more reactive oxygen species (ROS); high levels of ROS enhance the enzymatic activity of p38/MAPK and the expression of inflammation-related cytokines in macrophages, which accelerates inflammation and cancer transformation [74]. Furthermore, the signaling pathway formed by endothelial lipase through DTX3L-ISG15 also promotes the growth and metastasis of triple-negative breast cancer [75].

On the other hand, Yoo et al. demonstrated that ISGylation promotes the E3 ubiquitin ligase activity of Hsp70-interacting protein (CHIP), which subsequently reduces the level of oncogenic c-Myc, one of its many ubiquitination targets, and inhibits A549 cells and tumor growth [76]. Furthermore, CYP1B1 can inhibit the expression and covalent modification of ISG15 in the Hela cell line, preventing β-catenin degradation by ISG15 modification and activating the Wnt/β-catenin signaling pathway, resulting in abnormal cell proliferation and differentiation [77]. In lung cancer model mice, Mustachi et al. found that the elevated level of ISGylation could inhibit tumor growth by upregulating autophagy [78].

Taken together, protein ISGylation can promote cancer progression by modulating signaling pathways such as Ki-Ras, EGFR recycling, and Akt in breast cancer and is related to cell proliferation in prostate cancer and PDAC. In addition, the effect of protein ISGylation on lung cancer is dual; it can not only promote tumorigenesis and development by affecting the YAP-proteasome pathway but also inhibit tumor growth by affecting the ubiquitination and degradation of c-Myc (Table 2).

### 3.3. ISG15 Covalent Modification System and Cancer

#### 3.3.1. UBA7 and Cancer

**Figure 6:**
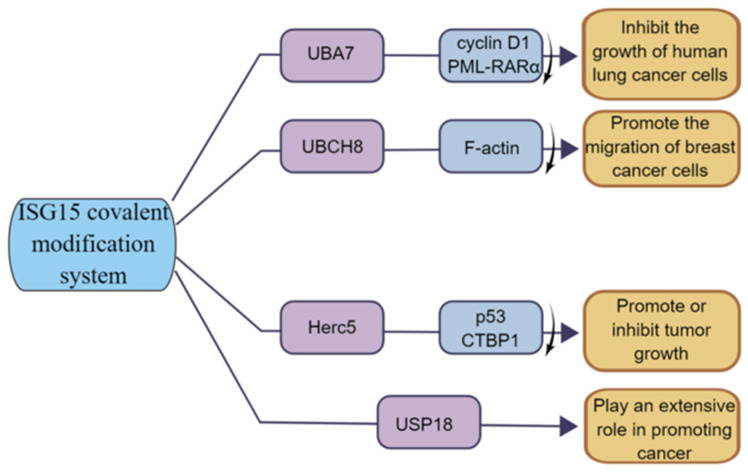
**The function of ISG15 modification system in tumor.** UBA7 can inhibit the growth of human lung cancer cells; UBCH8 can promote the invasion of breast cancer cells; HERC5 has both tumor-promoting and tumor-suppressing functions; USP18 has a positive role in promoting cancer.

The E1 enzyme UBA7 (also named UBE1L) of the ISG15 covalent modification system is located on chromosome 3p21, and 3p21 is missing in lung cancer patients; thus, UBA7 is often lowly expressed in lung cancer [81]. The high expression of UBA7 can target cyclinD1 and PML-RARα for proteasome degradation, thereby inhibiting the growth of lung cancer cells [82] (Figure 6). Moreover, studies have shown that TNF-α can directly upregulate the expression of UBA7 in cancer cells through the p38/MAPK and JNK pathways, and TNF-α is the earliest and most important inflammatory mediator which can activate neutrophils and lymphocytes, indicating the critical role of UBA7 in the inflammatory response related to cancer progression [83]. Therefore, UBA7 may be an important regulator of anti-cancer and inflammatory responses.

#### 3.3.2. UBCH8 and Cancer

The E2 enzyme UBCH8 (also known as UBE2E2) is currently less investigated in cancer. Some studies have shown that UBCH8 is expressed at a higher level in drug-sensitive cell lines compared to drug-resistant cells and can be expressed at a certain level during autophagy in esophageal cancer [84]. In breast cancer, UBCH8 promotes breast cancer cell migration by disrupting F-actin structure and promoting the formation of focal adhesion [69] (Figure 6). In addition, UHFR1 is highly expressed in HPV-positive cervical cancer cells, which negatively regulate UBCH8 by increasing the methylation on the *UBCH8* gene promoter, and inhibiting UHRF1 can upregulate the expression of UBCH8, thereby promoting UBCH8-induced apoptosis [85]. Thus, UBCH8 can regulate cancer cell migration and apoptosis to some extent.

#### 3.3.3. HERC5 and Cancer

As the E3 ligase of ISG15, HERC5′s function is closely related to ISGylation. For example, the E3 ligase HERC5 mediates the covalent modification of the tumor suppressor protein p53 by ISG15, enabling it to be degraded by the 20S proteasome [39]. Studies have shown that HERC5 is highly expressed in hepatocellular carcinoma tissues and cell lines, and researchers have screened a 7, 11-disubstituted quinazoline derivative, HZ-6d, which can bind to the HERC5 G-rich sequence and upregulate the expression level of the p53 protein, thus inhibiting tumor cell growth [80]. In breast cancer, a group of bioinformatics studying breast cancer patients found that HERC5 was associated with lymph node metastasis and tumor grade and significantly affected patients’ prognoses [86]. Furthermore, Lin et al. found that HERC5 level is downregulated in colorectal cancer (CRC), and downregulation of HERC5 attenuates the ubiquitination of CtBP1, which then accumulates and assembles into a complex with histone deacetylase 1 and the transcription complex of the transcription factor c-Myc. This transcriptional complex binds to the promoters of three pro-apoptotic genes, namely Bcl2-associated X (BAX), Bcl2-interacting killer (BIK), and p53-upregulated regulator of apoptosis (PUMA), and represses their expression, thereby inhibiting apoptotic signaling and promoting tumorigenesis. Overexpression of HERC5, downregulation of CtBP1, or blockade of CtBP1 function with its inhibitors (NSC95397 and 4-methylthio-2-oxobutyric acid [MTOB]) significantly prevents CRC cell proliferation in vitro and tumor growth in vivo [87] (Figure 6). The function of HERC5 in cancer is closely related to ISGylation, and the study of the role of HERC5 in cancer might be in coincidence with the change in ISGylation level.

#### 3.3.4. USP18 and Cancer

USP18 is also associated with tumorigenesis [88,89,90,91]. Studies have shown that USP18 expression is upregulated in various tumors, and loss of USP18 is involved in the destabilization of PTEN [73]. Cancer-specific survival was longer in the low USP18 expression group than that in the high expression group, suggesting that upregulated USP18 is an important risk factor for cancer-specific death [92]. Moreover, recent studies have shown that deletion of USP18 reduces tumor cell proliferation, migration, and invasion [93]. In addition, Xi et al. demonstrated that the cancer-promoting effect of USP18 may be achieved through the control of fatty acid metabolism in lung cancer [94] (Figure 6). Notably, some other studies have shown that USP18 has a strong tumor-promoting effect, indicating that USP18 functions through other important pathways besides de-ISGylase [62,78].

## 4. Clinical Applications of ISG15

ISG15 expression exhibits heterogeneity in tumors; in addition to a small number of cancers, ISG15 expression is obviously upregulated in a large number of tumors, such as bladder cancer, breast cancer, and ovarian cancer (Figure 3A). For example, the expression of ISG15 has been demonstrated to be elevated in esophageal squamous cell carcinoma (ESCC) and to be closely associated with clinical outcome, indicating that ISG15 may be used as a prognostic biomarker in ESCC patients [95,96]. In papillary thyroid microcarcinoma, ISG15 has been shown to be a prognostic marker in patients with lymph node metastasis [97]. These results suggest that ISG15 may become a biomarker as good as other biomarkers for tumor prognosis or diagnosis in ISG15-upregulated tumors. However, there are still no clinical applications concerning when ISG15 is downregulated, which should be further explored in the future.

The induction of ISG15 and ISGylation is associated with tumor treatment efficiency, including chemotherapy and radiotherapy [98,99]. As a result, elucidating the mechanisms by which ISG15 and its conjugation mediate sensitivity or resistance to cancer therapies could be a goal in improving cancer patient survival. Furthermore, some available data suggest that intracellular ISG15 conjugates and free ISG15 could harm patients by stabilizing cellular proteins that promote cancer, and secreted free ISG15 may benefit patients by modulating immune system functions [97]. It is suggested that targeting ISG15 alone is not a good method and that exploring its key substrate proteins and then targeting the ISGlylation of substrate proteins may be a better method. Thus, ISG15 clearly has double-edged functions in malignant cancers, and proper consideration must be given to assessing risk-benefit prior to administering ISG15-targeted cancer therapy to cancer patients when available.

## 5. Discussion and Conclusions

According to research, ISG15 is an interferon-stimulated gene, and the innate immune system can stimulate the production of ISG15 monomers and play an immunoregulatory role in tumors. In addition, ISG15 can also covalently modify some other proteins to regulate the occurrence and development of tumors. Both the monomeric and covalent modification functions of ISG15 are dysregulated in tumor tissues, while the covalent modification function could be the main factor. ISG15 exhibits the duality of tumor-promoting and tumor-suppressing in different tumor systems, indicating the complexity of the regulation of ISG15 in the process of tumorigenesis and development. By studying the regulatory role of ISG15 in tumors, we can deepen our understanding of tumorigenesis and development and provide new research ideas for finding ways and methods of tumor therapy. At present, the research on ISG15 is mainly focused on anti-viral infection and immune regulation, and its regulation on tumor function still needs further study. In general, free ISG15 acts as an immunomodulator as a cytokine in most cases, whereas ISGylation acts on oncoproteins to promote cancer in many cases. We speculate that the regulation of ISG15 and ISGylation in tumor cells is more like a secondary regulatory factor; its function depends more on the main factor, and the function of the main factor plays an important role in the regulation of ISG15 in tumor cells.

In conclusion, ISGylation is emerging as a key factor in cancer diagnosis, prognosis, and treatment strategies. However, it must be noted that free ISG15 has the potential to enhance the immune system, whereas ISGylation may contribute to pathology. Further investigation of the function of ISGylation and free ISG15 should be considered to assess risks and benefits before patients are offered ISG15-targeted cancer therapy, if available. Moreover, there is a dynamic balance between the content of free ISG15 and the content of covalently bound ISG15, and the level of ISGylation of intracellular molecules is likely to be affected by the content of intracellular and extracellular free ISG15 and further affect cell function. In addition, there is growing evidence that the level of protein ISGylation is likely to affect its ubiquitination, and the regulation of many molecules in cancer is regulated by its ubiquitination. In this regard, ISG15/ISGylation has great potential as a modulator of cancer therapy and clinical application.

In this review, we introduce the important relationship between ISG15/ISGylation and cancer and emphasize new insights into the different roles of ISG15/ISGylation in cancer progression. However, due to current research limitations, the regulation of ISG15/ISGylation in cancer is not completely clear. The current research is limited to the correlation between a certain tumor and ISG15/ISGylation or the impact of ISGylation of a molecule on the occurrence and development of tumors. Further comprehensive and sufficient correlation studies are urgently required, which leads to a lack of research on ISG15-related molecules as potential tumor therapy, and subsequent studies on the relationship between ISG15 and cancer can consider similar directions.

## Figures and Tables

**Table 1:** UBL proteins and their functions.

UBL Proteins	E1	E2	E3	Function
UFM1	UBA5	UFC1	UFL1	Ufmylation is involved in reticulophagy (also called ER-phagy)-induced in response to endoplasmic reticulum stress [11]. Ufmylation of TRIP4 regulates nuclear receptor-mediated transcription [12].
SUMO family	SAE1,SAE2	Ubc9	PIAS, CBX4	Regulating protein stability and the interaction between proteins and subcellular localization [13].
ISG15	UBA7	UBCH8	HERC5, TRIM25	Cancer-promoting or -suppressing; Antiviral infection [14].
NEDD8	NAE1	UBE2M, UBE2F	RBX1, RBX2, FBXO11, c-CBL, DCNL1-5, IAPs, RNF111, RNF111, TFB3, TRIM40	Regulating embryonic development, cell division and proliferation [15,16,17].
UBL3	-	-	-	Regulating MHCII and CD86 in human dendritic cells (DCs) and macrophages, regulating immune response [18].
ATG8	ATG7	ATG3	ATG5-12	Regulation of autophagy [19].
ATG12	ATG7	ATG10	-	Involving in autophagy vesicle formation [20].
FAT10	UBA6	UBE2Z	Parkin	Affecting the occurrence, progression and drug resistance of tumors [21,22].

**Table 2:** The function of ISGylation in different tumors.

Tumor Type	ISGylation Protein	Functions	References
Breast cancer	Ki-Ras	Promote cell proliferation, migration, and EMT; enhance cytotoxic T cell-mediated tumor killing;	[79]
Lung cancer	YAP, PTEN, CHIP	Promote the occurrence and development of tumors, inhibit A549 cells and tumor growth, and inhibit tumor growth by upregulating autophagy.	[71,73,76,78]
Cervical carcinoma	β-catenin	Inhibit abnormal cell proliferation and differentiation.	[77]
Prostate cancer	-	Promote cell proliferation.	[72]
Pancreatic cancer	-	Maintain stemness of tumor.	[41]
Colorectal cancer	-	Enhance colonic inflammation-associated tumor development.	[74]
Glioma	OCT4	Positively regulate glioma cell stemness.	[40]
Hepatocellular carcinoma	P53	Inhibit apoptosis.	[80]

## Data Availability

All data were presented in this manuscript.

## Abbreviations

Full name	Abbreviation
Ubiquitin-like	UBL
Interferon-stimulated gene 15	ISG15
Post-translational modifications	PTMs
Ubiquitin-like modifier-activating enzyme 7	UBA7
Ubiquitin-conjugating enzyme E2 E2	UBCH8
Post-translational ISG15 modification	ISGylation
Interleukin 1b	IL-1b
Cancer stem cell	CSC
Interferon	IFN
Pancreatic ductal carcinoma	PDAC
Yes-associated protein	YAP
Reactive oxygen species	ROS
Phosphatidylinositol 3,4,5-trisphosphate 3-phosphatase and dual-specificity protein phosphatase	PTEN
Muscle-invasive bladder cancer	MIBC
Protein kinase R	PKR
Bcl2-associated X	BAX
Bcl2-interacting killer	BIK
P53-upregulated regulator of apoptosis	PUMA
